# Bone Morphogenetic Proteins, Carriers, and Animal Models in the Development of Novel Bone Regenerative Therapies

**DOI:** 10.3390/ma14133513

**Published:** 2021-06-24

**Authors:** Nikola Stokovic, Natalia Ivanjko, Drazen Maticic, Frank P. Luyten, Slobodan Vukicevic

**Affiliations:** 1 Laboratory for Mineralized Tissues, School of Medicine, University of Zagreb, 10000 Zagreb, Croatia; nikola.stokovic@mef.hr (N.S.); natalia.ivanjko@mef.hr (N.I.); 2 Clinics for Surgery, Orthopedics and Ophthalmology, Faculty of Veterinary Medicine, University of Zagreb, 10000 Zagreb, Croatia; drazen.maticic@vef.hr; 3 Skeletal Biology & Engineering Research Center, KU Leuven, Herestraat 49, 3000 Leuven, Belgium; frank.luyten@kuleuven.be

**Keywords:** animal model, bone fracture, bone healing, posterolateral spinal fusion, regenerative medicine, bone morphogenetic proteins

## Abstract

Bone morphogenetic proteins (BMPs) possess a unique ability to induce new bone formation. Numerous preclinical studies have been conducted to develop novel, BMP-based osteoinductive devices for the management of segmental bone defects and posterolateral spinal fusion (PLF). In these studies, BMPs were combined with a broad range of carriers (natural and synthetic polymers, inorganic materials, and their combinations) and tested in various models in mice, rats, rabbits, dogs, sheep, and non-human primates. In this review, we summarized bone regeneration strategies and animal models used for the initial, intermediate, and advanced evaluation of promising therapeutical solutions for new bone formation and repair. Moreover, in this review, we discuss basic aspects to be considered when planning animal experiments, including anatomical characteristics of the species used, appropriate BMP dosing, duration of the observation period, and sample size.

## 1. Introduction

Bone tissue possesses unique regenerative properties, and bone fractures regularly heal under physiological conditions. However, large segmental bone defects resulting from severe trauma or extensive tumor resection cannot be restored by endogenous self-repair mechanisms, decrease quality of life, and may sometimes lead to limb amputation. Indeed, the management of large segmental defects is one of the most challenging issues in orthopedic medicine, typically due to the biologically hampered microenvironment [1,2]. The standard of care for the healing of large bone defects requires the use of an autologous bone graft (ABG), which is usually harvested from the iliac crest. ABG is also used as a gold standard to achieve spinal fusions, including posterolateral spinal fusion (PLF). PLF is a commonly performed surgical procedure used for the treatment of degenerative diseases of the spine, including degenerative disc disease, spondylolisthesis, spinal instability, and symptomatic scoliosis [3,4,5,6].

However, ABG possesses several disadvantages, including a limited amount of bone that might be harvested, the potential transfer of contaminating agents, acute and chronic pain, skin scarring, and deformity at the donor site [4,7]. In addition, the use of ABG increases the blood loss, duration, and cost of surgical procedures. Therefore, there remains an imminent need for the development of novel bone regeneration strategies to enrich or replace ABG. Among these, osteoinductive devices are under investigation for clinical use in PLF and healing of large segmental long bone defects.

In the last few decades, numerous preclinical studies using animal models have been conducted to test novel bone bridging or fusion strategies [8,9]. The principles of the rational use of animal models in the evaluation of novel bone regenerative therapies have been previously described [8]. Hence, we further investigated the use of animal models in the development of osteoinductive therapies of large segmental bone defects and PLF procedures, in particular the selection of a proper anatomical model, treatment dose, observation period, and sample size. We specifically analyzed published in vivo studies looking into the development of bone morphogenetic protein (BMP)-based bone inducing implants.

## 2. Bone Regeneration by Bone Morphogenetic Protein Devices

BMPs are well-known osteoinductive molecules, required and sufficient for ectopic bone induction, and powerful agents for the restoration of large orthotopic bone defects [10]. BMP2 is the most widely used osteoinductive BMP in preclinical testing, and it is a part of an osteoinductive device (Infuse™, Medtronic, Dublin, Ireland), currently approved for anterior lumbar interbody fusion (ALIF), acute tibial fractures, and maxillofacial reconstructions [11,12,13,14,15]. However, BMPs have been used off- label in various spinal indications, including cervical spine fusion, posterior lumbar interbody fusion (PLIF), transforaminal lumbar interbody fusion (TLIF), posterolateral spinal fusion (PLF), and thoracolumbar fusions [16,17]. Reported side effects in patients included implant displacement, infection, swelling of the adjacent tissue and dysphagia, formations of seroma, radiculitis and nerve root compression, ectopic bone formation, osteolysis, and retrograde ejaculation [11,12,16,17,18,19,20,21]. These side effects eventually resulted from the use of supraphysiological doses as registered BMP2-based devices contain 4–12 mg recombinant protein, while the human body contains only a total of 2 mg of BMPs [22].

Other commonly used osteoinductive BMPs are BMP7, which is no longer in clinical use, and more recently, BMP6 [23]. We demonstrated that BMP6 appears to be superior to BMP2 and BMP7 in promoting osteoblast differentiation in vitro and inducing bone formation in vivo [23,24]. The superiority of BMP6 may arise from its resistance to noggin inhibition and affinity across the BMP type I receptors. Therefore, BMP6-based devices are expected to be more efficacious at lower doses compared to BMP2 and BMP7.

BMPs require a carrier/delivery system that will sustain the BMP concentration and allow prolonged BMP release [25,26,27,28]. Moreover, the ideal BMP carrier should be biocompatible, enable vascular and cellular infiltration, resist compression, and define the contours of the resulting bone [25,26,29]. BMP carriers can be divided into four major groups: natural polymers, synthetic polymers, inorganic materials, and combinations between these groups [25,26,30].

Natural polymers include collagen, hyaluronic acid, gelatin, fibrin, chitosan, alginate, and silk and have been extensively evaluated in preclinical studies [6,31,32,33,34,35,36,37,38]. The advantages of natural polymers are biocompatibility, biodegradability, and resorbability in the physiological environment [25,26,27]. The most commonly used is bovine tendon collagen which delivers BMP2 in the clinically approved Infuse™ device. However, collagen has significant disadvantages, including a low affinity for BMPs, immunogenicity due to its animal origin, and weak biomechanical properties resulting in compression by surrounding tissues [25,26,27].

Biocompatible and biodegradable synthetic polymers, such as polylactic acid (PLA), polyglycolic acid (PGA), poly(D, L-lactide-co-glycolide) (PLGA), polyethylene glycol (PEG), poly-E-caprolactone (PCL), and polypropylene fumarate (PPF), as well as their block polymers have been evaluated as potential BMP carriers to overcome the disadvantages of natural polymers, including immunogenicity and disease transmission risk [39,40,41,42]. They are also moldable into highly porous three-dimensional scaffolds, linearly oriented scaffolds, fibers, sheets, blocks, or microspheres [26]. Apart from these advantages, synthetic polymers decrease local pH as a result of acidic breakdown byproducts, have poor clearance, cause bulk degradation, and cause chronic inflammation associated with high-molecular weight polymers, resulting in substantial disadvantages [26]. They have also been tested with other potentially osteogenic molecules, such as PGE2 and PGE4 prostaglandin receptor analogs [43], and materials such as calcium silicate (CaSi) and dicalcium phosphate dihydrate (DCPD) [44,45,46,47].

Inorganic materials as potential BMP carriers include calcium phosphate (CaP) ceramics, calcium phosphate and calcium sulfate cement, and bioglass [2,5,29,32,38,42,48,49,50,51,52,53,54,55,56,57,58,59,60,61,62,63,64,65,66,67,68,69,70,71,72]. The most commonly used inorganic preclinical materials are CaP ceramics, further subdivided into hydroxyapatite (HA), tricalcium phosphate (TCP), and biphasic calcium phosphate (BCP) containing both HA and TCP at various ratios. We have recently shown that the chemical composition of ceramics does not affect the amount of newly formed bone induced by the osteoinductive device [73,74]. However, HA and TCP significantly differ in resorbability (HA is very stable, while TCP is more resorbable), which would eventually result in different residual ceramic volumes. The resorbability might be adjusted by varying HA/TCP ratios in BCP ceramics [75]. Moreover, CaP ceramics might be formulated into particles or blocks in a broad range of sizes and geometrical shapes while porosity, pore size, and interconnectivity are adjusted during the sintering process [73,75,76]. We demonstrated that particle size affects the volume of newly formed bone; smaller particles (74–420 µm) combined with rhBMP6 resulted in higher bone volume than larger particles (1000–4000 µm) [73]. Another important determinant of ceramics is the pore size since pores from 300 to 400 µm promoted the formation of the largest bone volume [51].

The fourth group of BMP carriers are composites of the aforementioned materials which have been introduced to overcome the encountered limitations of a single component. The most typical combinations are composites containing either natural or synthetic polymers with CaP ceramics [39,77,78,79,80,81,82,83,84,85]. In these combinations, ceramics increase the biomechanical properties of the implants and are used to address compressibility issues. Less frequent, natural, and synthetic polymers might be combined.

We have recently developed an autologous bone graft substitute (ABGS) comprised of BMP6 delivered within an autologous blood coagulum to which a compression-resistant matrix, such as allograft or synthetic ceramics, can be added [22,73,74,76,86,87,88,89,90,91,92]. Moreover, the volume of newly induced bone increased with the elevation of the CRM amount, which might be attributed to the enlargement in an overall surface area [73].

## 3. Animal Models

**Figure 1:**
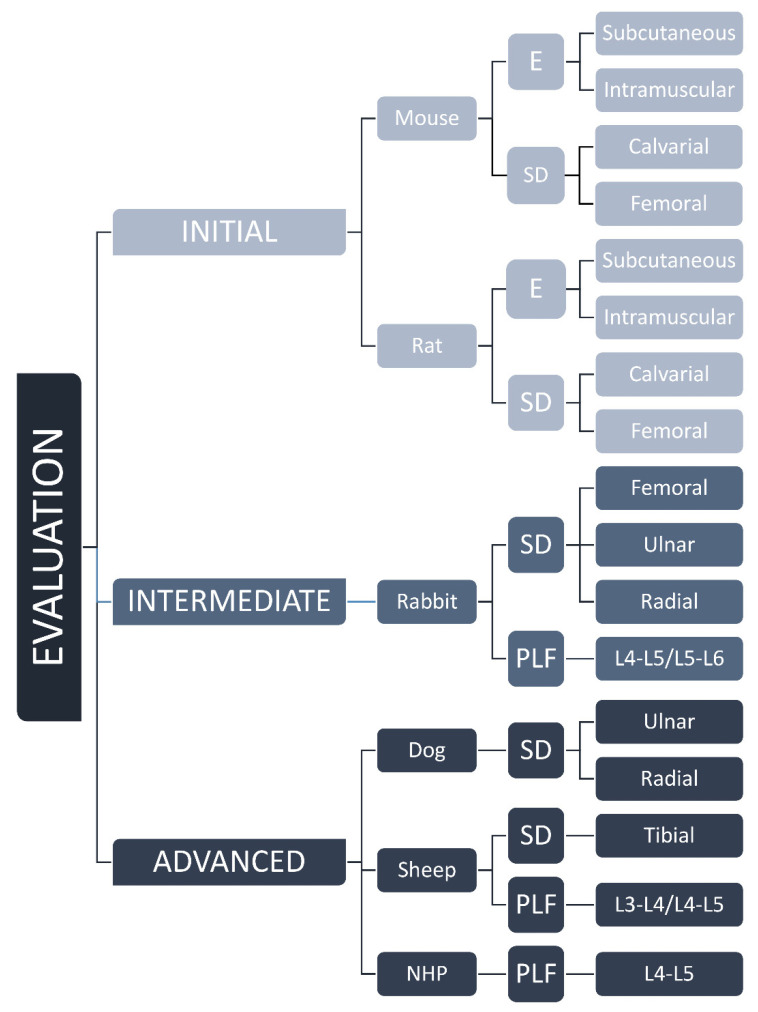
Classification of animal models from mouse to NHP based on the stage of preclinical development, namely as initial, intermediate, and advanced evaluation of osteoinductive devices. E—ectopic, SD—segmental defect, PLF—posterolateral spinal fusion models, and L—level of lumbar transverse processes.

Animal models are routinely used in the development of novel bone regenerative therapies [8]. Models might be categorized according to the species (mouse, rat, rabbit, sheep, non-human primate) and tested indication (ectopic model, critical-size defect, PLF). In this review, we suggested classification based on the stage of preclinical development, namely as initial, intermediate, and advanced testing of osteoinductive devices (Figure 1). Initial testing includes rodent ectopic and rodent critical-size defect models for rapid comparison of different osteoinductive responses. Intermediate evaluation includes adequate rabbit models (segmental defect and PLF model), while advanced testing uses canine, sheep, and non-human primates as a final step before clinical trials.

### 3.1. Initial Evaluation in Rodents

#### 3.1.1. Ectopic Models

**Figure 2:**
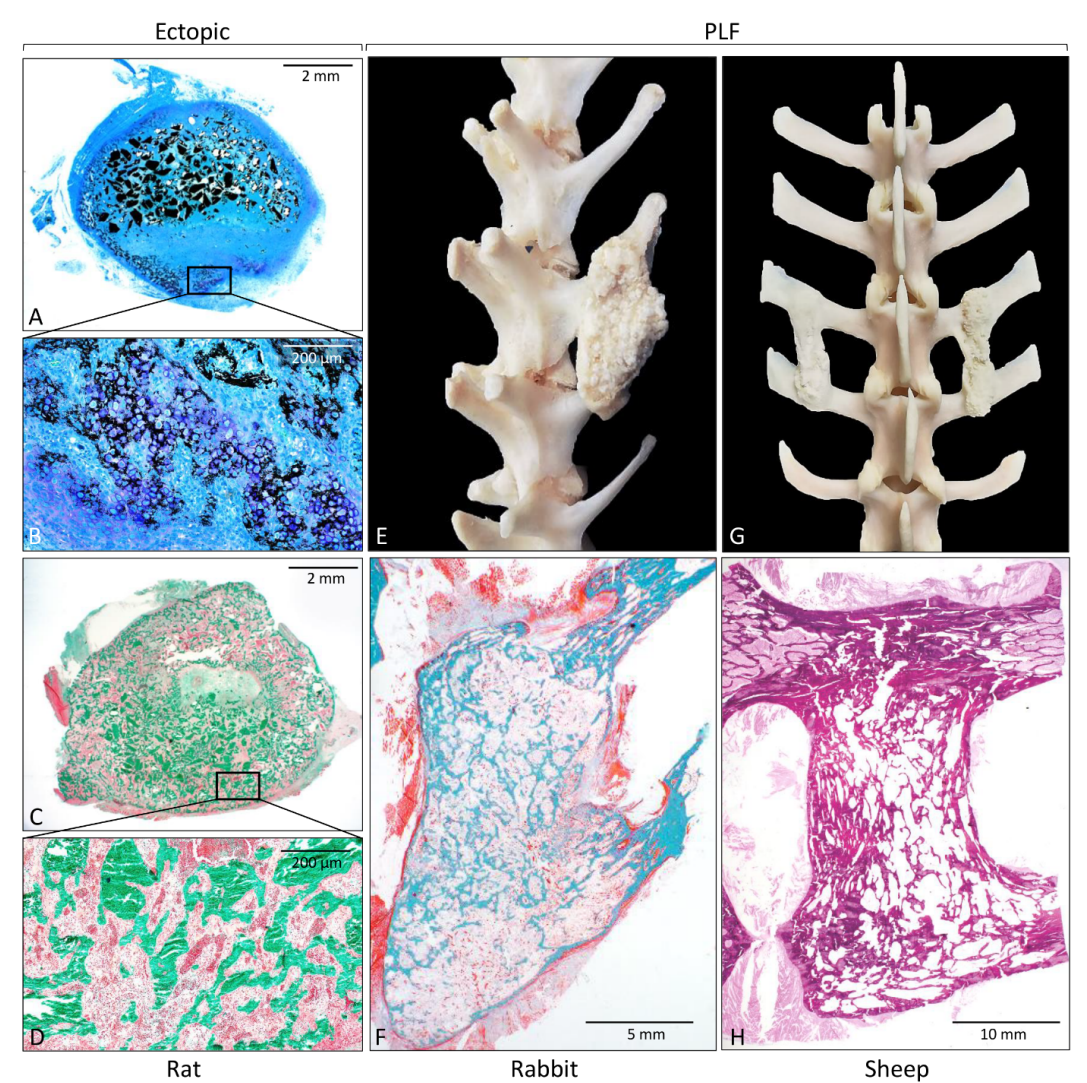
Histological sections and gross anatomy of newly formed bone induced by rhBMP6 in a rat subcutaneous assay (**A**–**D**); rabbit (**E**,**F**) and sheep (**G**,**H**) posterolateral spinal fusion model. (**A**,**B**) On day 7 following implantation, endochondral bone formation occurs at the peripheral site of ABGS, while 14 days (**C**,**D**) after implantation, newly formed bone is present throughout the implant containing rhBMP6 lyophilized on allograft mixed with ABC. Gross anatomy of newly formed bone between transverse processes in rabbit (**E**) and sheep (**G**) PLF model. Histological sections through fusion mass in rabbit (**F**) and sheep (**H**) PLF model. Histological sections were processed undecalcified and stained by Von Kossa (**A**,**B**), Goldner (**C**,**D**,**F**), and hematoxylin and eosin (**H**). (Modified from [74,88], respectively.)

Rodent ectopic models have been extensively used for the initial evaluation of novel osteoinductive therapies. They might be also used for investigating the biology of ectopic bone induction and the formation of a bone organ or ossicle, including bone and bone marrow [31,32,39,48,49,50,51,52,53,54,55,56,57,71,73,76,86,87,93,94,95,96,97,98,99,100,101,102,103,104,105]. Rodent ectopic models (Table 1 and Table 2) are further subdivided according to the species (mouse, rat) and the implantation site (subcutaneous or intramuscular). Implantation under the skin (Figure 2A–D) or into the muscle does not affect the bone formation outcome, and the bone formation occurs in the first two weeks following implantation of an osteoinductive device [76,86,87]. The later time points are needed for the evaluation of the bone longevity and maintenance of the ectopic bone structure. Molecular and cellular events during the cascade of bone formation can be evaluated using microCT/nanoCT and histological analyses. Immunohistochemistry, flow cytometry, gene profile microarrays, and single-cell RNA sequencing are among other analytical techniques used for unraveling the mechanism of ectopic bone formation.

Murine models are initially used to unravel the potential mechanism of action of various signaling pathways and genes or proteins in bone induction or enhancement. However, due to limited translation of mouse to human bone regeneration and disease outcome, the rat is therefore a more suitable model when testing functional outcomes [106,107,108].

#### 3.1.2. Bone Defect Models

**Figure 3:**
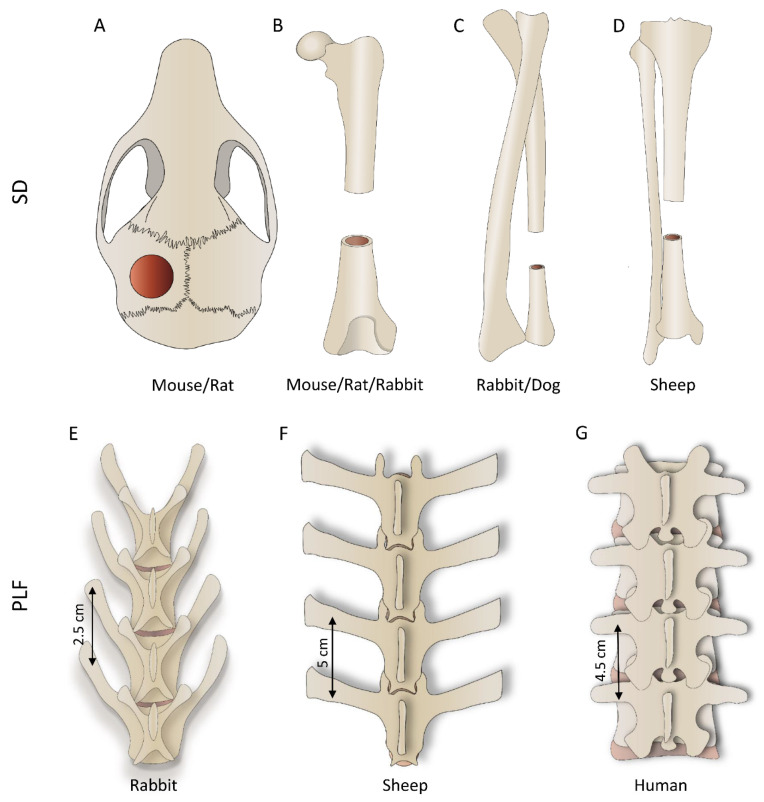
First row: segmental defect (SD) models performed on various bones depending on the species; (**A**) calvarial, (**B**) femoral, (**C**) ulnar (or radial), and (**D**) tibial defect. Second row: posterolateral lumbar fusion (PLF) is conducted between adjacent transverse processes; the figure shows differences in the anatomy of rabbit (**E**), sheep (**F**), and human (**G**) lumbar spine.

**Figure 4:**
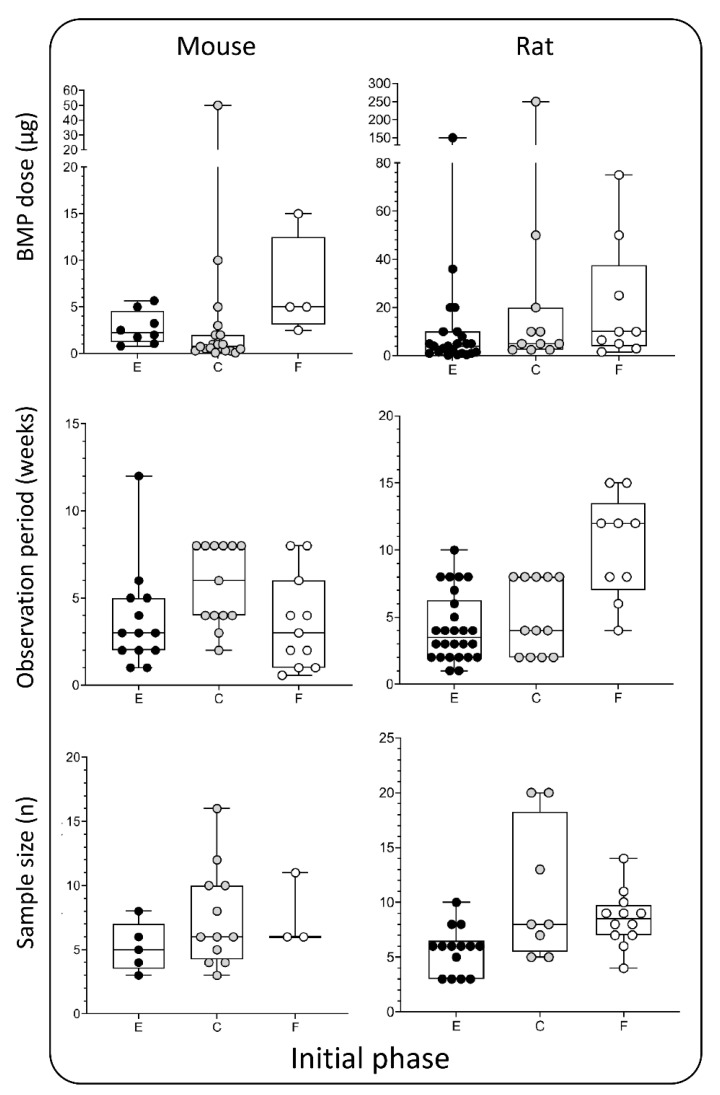
BMP doses (µg), observation period (weeks), and sample size (*n*) used in the initial phase —rodent models. Data is presented through box and whisker plots in which dots represent all individual values from studies listed in Table 1 and Table 2. E—ectopic model, C—calvarial, and F—femoral defect.

Mouse or rat bone defects are the initial orthotopic models to evaluate the osteoinductive properties of novel therapies and the osseointegration of newly formed bone with adjacent native bone. There are two main bone defect models in rodents: a calvarial critical-size defect and segmental femoral defect. In the calvarial critical-size defect, circular bone defects are created in the mouse (3–5 mm) [109,110,111,112,113,114,115,116,117,118] and rat (4–8 mm) [29,55,58,59,60,66,77] calvaria (Figure 3A; Table 1 and Table 2), while segmental defects of the long bone are typically created in the femur, both in mice (2–3 mm) [119,120,121] and rats (6–10 mm) [5,33,34,35,67,78,79] (Figure 3B; Table 1 and Table 2) and filled with tested osteoinductive material. The development of a reproducible non-union model in the mouse is demanding, and, in contrast to rat non-union models, mouse non-union models are sparse [122]. The main shortcoming of this model is a relatively small defect size compared to clinically relevant proportions. Moreover, it is difficult to obtain a full stabilization of the fracture, therefore resulting in increased callus formation. Methods of evaluation include analyses of radiological images (CT/microCT), histological and histomorphometric analyses, and biomechanical testing, which might be conducted at the end of the observation period (Figure 4) [5,29,33,34,35,55,58,59,60,66,67,77,78,79,109,110,111,112,113,114,115,116,117,118,119,120,121,123].

### 3.2. Intermediate Evaluation in Rabbits

#### 3.2.1. Segmental Defect Model

**Figure 5:**
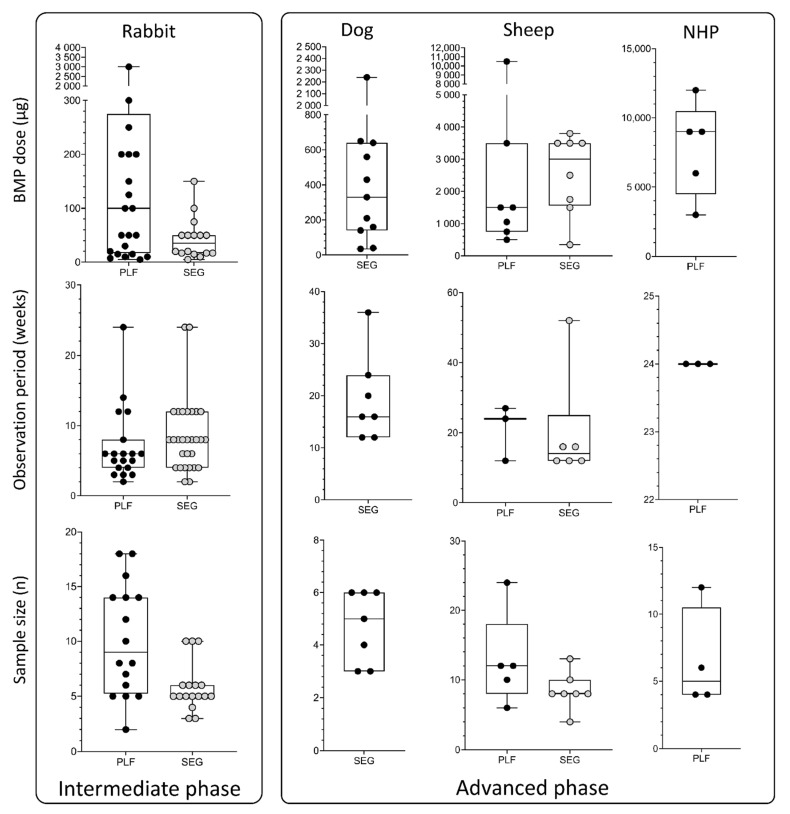
BMP doses (µg), observation period (weeks), and sample size (*n*) used in the intermediate and advanced phases, which include rabbit, dog, sheep, and non-human primate models. Data are presented through box and whisker plots in which dots represent all individual values from studies listed in Table 3, Table 4, Table 5 and Table 6. SEG—segmental defect models; PLF—posterolateral spinal fusion model.

Potential therapeutical solutions for segmental bone defect restoration have been extensively evaluated in rabbits [36,41,42,61,68,69,70,80,81,82,83,84,87,124,125,126] (Table 3), and a defect can be created in the femur, radius, or ulna (Figure 3B,C). Regardless of the chosen anatomical site, the typical defect size is 15–20 mm. In published work, the defect was bridged with a broad range of delivery systems containing up to 150 µg of BMPs. The observation period was typically 6–12 weeks. Few studies evaluated bone formation at earlier time points (2 and 4 weeks) or for a prolonged period (24 weeks) (Figure 5, 1st column).

#### 3.2.2. Posterolateral Spinal Fusion (PLF) Model

Rabbit is the most commonly used species for the evaluation of the efficacy and safety of promising therapeutical solutions for achieving PLF [5,6,37,38,40,62,63,64,74,85,86,127,128,129]. The transverse processes of lumbar vertebrae are exposed, and an osteoinductive device is implanted bilaterally between adjacent transverse processes (L4-L5 or L5-L6) [127]. Transverse processes should be decorticated before the implantation to promote osseointegration of newly formed bone with native bone [86]. In the majority of previous studies, the BMP dose was up to 1000 µg and was delivered on various carriers (Table 4). The spinal fusion outcome was evaluated 6 weeks following surgery, and the majority of rabbit PLF studies had an observation period of fewer than 10 weeks. Few studies had a prolonged observation period (>10 weeks), but later time points might be important to determine the survival and long-term maintenance of newly induced bone [6,86], which is clinically relevant in patients undergoing PLF surgery (Figure 5, 1st column).

Methods of evaluation in rabbit segmental defect and PLF models are similar and include segmental mobility testing, radiological methods (x-ray and CT/microCT), histological (Figure 2F), histomorphometric analyses, and biomechanical testing [5,6,37,38,40,62,63,64,74,85,86,127,128,129].

### 3.3. Advanced Evaluation of Bone Regeneration Therapies

#### 3.3.1. Dog and Sheep Segmental Defect Model

Dog and sheep segmental defect models are used for advanced evaluation of bone regeneration therapies. In dogs, the defect (20–25 mm) is created in the radius or ulna (Figure 3C) [130,131,132,133,134,135]. Applied doses of BMPs were in the range between 100 and 650 µg, which is higher compared to the rabbit model. Moreover, the typical observation period (12–24 weeks) was also prolonged compared to the rabbit model (Figure 5, 2nd and 3rd columns).

Tibial segmental bone defects in sheep (Figure 3D) were recently developed to evaluate novel bone regeneration therapies in conditions mimicking the size and biology of segmental bone defects in the clinics [2,136,137,138,139,140]. Moreover, there are two subtypes of this model: a fresh defect (FD) and biologically exhausted defect (BED), the latter mimicking a patient with a non-union [2]. Following the creation of a large defect (30 or 45 mm) in the sheep tibia in the FD model, a polymethyl-methacrylate spacer is inserted to induce the formation of the Masquelet membrane. Six weeks following the creation of the defect, an osteoinductive device was inserted after the removal of the spacer (FD model). In the BED model, the defect is in the first instance left untreated leading to a non-union. Subsequently, debridement of the non-union or fibrotic tissue ingrowth (BED model) is performed, followed by implantation of a spacer for 6 weeks, and then, finally, after removal of the spacer, an implant is inserted. BMP doses applied in this model ranged from 344 to 3800 µg, while the typical observation period was up to 16 weeks [2]. Although the osteoinductive device containing BMP6 on a carrier achieved bridging in FD (30 mm), it was found that larger, biologically exhausted defects appear to require a cell-based implant together with BMP to achieve proper clinically relevant bridging (Table 5) [2]. Importantly, defects were mechanically well stabilized with a circular external fixator according to the Ilizarov technique.

#### 3.3.2. Sheep PLF Model

The sheep PLF model is highly translatable to clinics because the size of the lumbar vertebrae of the sheep is comparable to humans. However, only a few preclinical studies have been conducted on this model [3,65,88] (Table 6). Sheep PLF may be conducted at a single level or as a multisegmental procedure. Moreover, it may be performed with or without instrumentation [88]. The observation period and applied BMP doses in this model were typically significantly longer/higher than in studies on small animals: the follow-up period was up to 6 months with a BMP amount up to 10 mg (Figure 5, 3rd column). Methods of evaluation included X-ray monitoring, microCT evaluation, histological analyses (Figure 2H), and biomechanical testing [3,65,88].

#### 3.3.3. Non-human Primate (NHP) PLF Model

Non-human primates are the most similar animal species to humans, both anatomically and genetically. However, only a few studies were conducted using NHP PLF [14,63,141] (Table 6), primarily due to ethical and economic reasons. In these studies, the goal was to achieve a single-level fusion between adjacent lumbar transverse processes, which are anatomically similar to humans. The applied BMP2 doses (3–12 mg), as well as observation period (24 weeks), were comparable to the sheep PLF model (Figure 5, 4th column).

### 3.4. Anatomical Characteristics of the Species

#### 3.4.1. Segmental Bone Defect

The general anatomy of long bones (Figure 3B–D) of species discussed in this review is similar, and obviously, the greatest difference among them is size. Differences in the bone size reflect the segmental defect created in each species. The length of the long bone segmental defect in mice (3 mm) or rats (5–8 mm) is small and created in the femur, the largest bone in rodents [5,33,34,35,67,78,79,119,120,121,123,142]. Long bones are significantly larger in rabbits/dogs, and segmental defects (15–20 mm in rabbits and 20–25 mm in dogs) are created in the femur, radius, or ulna [36,41,42,61,68,69,70,80,81,82,83,84,87,124,125,126,130,131,132,133,134,135]. Sheep segmental bone defects are usually created in the tibia and, due to their size (30–45 mm), are considered clinically relevant because the defect size compares to those in patients [2].

#### 3.4.2. PLF

Posterolateral spinal fusion (PLF) in preclinical studies is conducted in the lumbar portion of the spine. Although the basic anatomical features of lumbar vertebrae are similar among species discussed in this review, they differ in size and proportions of the different parts of the vertebrae. Rabbits (Figure 3E) and sheep (Figure 3F) have long transverse processes compared to the size of the vertebral body, while humans (Figure 3G), as an adaptation to erect posture and bipedal locomotion, have large bodies and short transverse processes. Importantly, transverse processes in rabbits are slanted and oriented anteriorly (Figure 2E or Figure 3E). On the other hand, the transverse processes in sheep (Figure 2G or Figure 3F) and humans (Figure 3G) are horizontal. The distances between the transverse processes are relatively short in rabbits (20–30 mm), while they are comparable in sheep and humans (40–50 mm).

## 4. Appropriate Bone Morphogenetic Proteins Dosing

The selection of the appropriate dose for each indication is one of the most challenging steps in the design of experiments. In this review, we compared doses used in various models in mice, rats, rabbits, sheep, and NHP. In the mouse and rat ectopic models, BMP doses were typically up to 25 µg [31,32,39,48,49,50,51,52,53,54,55,56,57,71,73,76,86,87] (Figure 4, 1st column, 1st row) per implant, while they were increased in the rat bone defects (up to 50 µg) [5,29,33,34,35,55,58,59,60,66,67,77,78,79,123] (Figure 4, 2nd column, 1st row). BMP doses used in rabbits were up to 150 µg in segmental bone defects [36,41,42,61,68,69,70,80,81,82,83,84,87,124,125,126,143] (Figure 5, 1st column, 1st row) and up to 300 µg in rabbit PLF procedures [5,6,37,38,40,62,63,64,74,85,86,127,128,129] (Figure 5, 1st column, 1st row). Tested BMP doses in dogs were higher and were typically in the range between 100 and 650 µg [130,131,132,133,134,135] (Figure 5, 2nd column, 1st row). Moreover, in the sheep and NHP models, BMP doses were significantly higher: in the sheep, the doses were between 500 µg and up to 4 mg [3,65,88] (Figure 5, 3rd column, 1st row), while in the NHP, the doses were up to 12 mg [14,63,141] (Figure 5, 4th column, 1st row).

## 5. Duration of the Observation Period

The bone induction process is significantly faster in small animals compared to higher species animals and humans. Therefore, observation periods were significantly shorter in rodent ectopic and bone defect models than in studies on sheep and non-human primates (Figure 4 and Figure 5, 2nd row). The average observation period in mouse and rat ectopic models was 3–4 weeks [31,32,39,48,49,50,51,52,53,54,55,56,57,71,73,76,86,87] (Figure 4, 1st and 2nd columns, 2nd row). However, depending on the purpose of the study, observation periods in these studies might vary from a few days (studies on the mechanism of bone induction) to several months (bone longevity). Typical observation periods in the rat calvarial defect and femoral segmental defect models are slightly prolonged and last 5 and 10 weeks, respectively [5,29,33,34,35,55,58,59,60,66,77,78,79,123] (Figure 4, 2nd column, 2nd row). The observation period in segmental defect studies was up to 12 weeks in rabbits. [36,41,42,61,68,69,70,80,81,82,83,84,87,124,125,126,130,131,132,133,134,135,143] (Figure 5, 1st column. 2nd row). On the other hand, spinal fusion outcome in the rabbit PLF model was typically evaluated after 6 weeks [5,6,37,38,40,62,63,64,74,85,86,127,128,129] (Figure 5, 1st column, 2nd row). However, to study longevity or resorbability of compression-resistant matrices, the follow-up period might be prolonged. As expected, a longer observation period in dogs and sheep was up to 12 months [3,65,88,130,131,132,133,134,135] (Figure 5, 2nd and 3rd columns, 2nd row), while in NHP studies, it was 6 months [14,63,141] (Figure 5, 4th column, 2nd row).

## 6. Sample Size

Defining an appropriate sample size is a prerequisite for obtaining valid conclusions from each study. Moreover, the appropriate size of the sample is affected by several parameters, including experimental design and purpose of the study as well as expected differences among experimental groups. The sample size in the majority of reviewed studies here was 5–10 per group regardless of the animal species or model (Figure 4 and Figure 5, 3rd row). Moreover, there is a consensus in published work that the minimal number of animals per experimental group is four. However, a few animals might die during surgery or follow-up periods due to reasons non-related to the tested osteoinductive therapy; therefore, at least five animals per experimental group should be included.

## 7. Study Outcomes

In Table 1, Table 2, Table 3, Table 4, Table 5 and Table 6, it was not possible to describe the study outcomes due to non-comparable scoring grades for healing or spinal fusion experiments. The prerequisite in reporting the outcome of bone defect and spinal fusion studies is a clearly described success rate as the percentage of successfully rebridged defects or fused spine segments, respectively. Moreover, the method (radiological images, mobility testing) used to determine rebridgment/fusion should be clearly described. Surprisingly, in a large number of published studies, the success rate was not explicitly described. Several authors used their own scoring grades instead of standardized binary outcomes (successful or unsuccessful rebridgment/fusion). However, even when the binary outcome was used, the determination of successful rebridgment/fusion differed among authors. For example, a few authors determined success rate only on X-ray images without microCT, histology, and biomechanical testing. We suggest that successful rebridgment/fusion should also be determined with microCT, histological sectioning, and biomechanical testing.

The experimental outcome of osteoinductive therapies using rodent ectopic models should be determined by microCT and histology. MicroCT analyses provide information on newly formed bone volume expressed as bone volume (BV) or bone volume/tissue volume ratio (BV/TV). Additionally, if the tested osteoinductive device contains ceramics, microCT analyses might be used to determine the amount of residual ceramic matrix. Moreover, microCT analyses allow the determination of structural properties of newly formed bone by calculating trabecular parameters (trabecular number, trabecular thickness, trabecular separation). The structural properties of newly induced bone should also be analysed by histology and histomorphometry to determine the volume of the bone and remaining carrier/matrix.

## 8. Conclusions

Due to the large socioeconomic burden of degenerative diseases of the spine and segmental defects of long bones, there is an imminent need for the development of novel osteoinductive therapeutic solutions [1,22]. However, until now, none of the osteoinductive devices have been approved for use in PLF and large segmental defects in patients. A broad range of bone regeneration strategies have been proposed and tested in different animal models. A vast majority of these studies have been conducted in rats and rabbits, leading only to the initial and intermediate steps of preclinical testing, and despite claiming positive results, only a few have been further tested in sheep and NHP models. Infuse™, a BMP2-containing osteoinductive device, has been approved for use in ALIF and acute tibial fractures but has also been used in various off-label indications. However, numerous side effects related to high BMP dose and a large release from the bovine collagen as a carrier have been reported. Therefore, there is a need for an osteoinductive device that would be efficacious at lower doses of BMP delivered on a carrier with a prolonged BMP release. There is some hope that novel engineered BMPs or innovative delivery systems for BMPs may reduce the required therapeutic doses. A novel ABGS containing rhBMP6 within autologous blood coagulum was evaluated in preclinical studies, and in exploratory clinical trials (high tibial osteotomy, distal radial fracture, and posterolateral interbody fusion), it was proven safe and efficacious at relatively low BMP6 doses [73,74,76,86,87,88,91,92].

## Figures and Tables

**Table 1:** Mouse ectopic and bone defect models.

Mouse Ectopic Model					
Author (Year)		Carrier	BMP Dose (µg)	Time (Weeks or Days)	Sample Size (*n*)
Kato et al. (2006)		PLA-DX-PEG, PLA-DX-PEG/TCP, TCP	2 and 5	3 and 6 weeks	6
Roldan et al. (2010)		BCP		12 weeks	8
Liang et al. (2014)		TCP	50	3, 7, 14, 21, and 28 days	5
Bolander et al. (2016)		CaP granules/Collagen	1.06 and 1.77	5 weeks	4
Ji et al. (2018)		CaP-based materials	0.81, 3.24, and 5.67	2 and 5 weeks	3
Hashimoto et al. (2020)		Collagen	2.5	7, 10, 14, and 21 days	
**Mouse Calvarial Defect Model**					
**Author (Year)**	**Calvarial Defect Size (mm)**	**Carrier**	**BMP Dose (µg)**	**Time (Weeks)**	**Sample Size (*n*)**
La et al. (2012)	4	TCP, Heparin—conjugated fibrin	0.3	8	10
Yang et al. (2012)	4	Collagen, Apatite—coated collagen	0.5, 0.75, 1, 2, and 3	8	6
Fan et al. (2015)	3	PLGA/Apatite layer	0.3, 0.6, and 1	6	8, 12
Gronowitz et al. (2017)	3.5	Collagen/HA	2	3	4
Herberg et al. (2017)	5	Acellular dermis	0.542	4	10
Huang et al. (2017)	3.5	PLA	50	2, 4, 6, and 8	16
Seo et al. (2017)	5	Poly(phosphazene) hydrogels Poly(phosphazene) hydrogels/BCP	5 and 10	8	3
Terauchi et al. (2017)	3.5	Sulphopropyl ether—modified polyrotaxanes/Collagen	0.1	4	5, 6
Maisani et al. (2018)	3.5	Hydrogel	1	8	6
Reyes et al. (2018)	4	PLGA	0.1, 0.3, and 0.6	4 and 8	4
**Mouse Femoral Defect Model**					
**Author (Year)**	**Femoral Defect Size (mm)**	**Carrier**	**BMP Dose (µg)**	**Time (Weeks)**	**Sample Size (*n*)**
Alaee et al. (2014)	2	Collagen	5	4 days, 1, 2, 3, 4, and 8	6
Bougrouli et al. (2016)	2	Collagen	5	1, 2, 4, and 8	6
Zwingenbergen et al. (2016)	3	Heparin/functionalized mineralized collagen matrix	2.5 and 15	6	11

**Table 2:** Rat ectopic and bone defect models.

Rat Ectopic Model				
Author (Year)	Carrier	BMP Dose (µg)	Time (Weeks)	Sample Size (*n*)
Kuboki et al. (1998)	HA		1, 2, 3, and 4	
Tsuruga et al. (1998)	HA	4	1, 2, 3, and 4	3
Alam et al. (2000)	TCP, HA, BCP	1.5 and 10	2 and 4	3
Vehof et al. (2002)	HA	8	3, 5, 7, and 9	3
Kim Chang-Sung et al. (2004)	TCP, Collagen	5	2 and 8	10
Tazaki et al. (2006)	HA	0.5, 1, and 5	3	
Tazaki et al. (2008)	HA, TCP	0.5, 1, and 5	3	3
Luca et al. (2010)	Chitosan/Hyaluronan hydrogel	150	3	3, 6
Reves et al. (2011)	Chitosan-nano-HA	36	4	6
Park et al. (2011)	BCP	2.5	2 and 8	5–8
Bhakta et al. (2012)	Hyaluronan-based hydrogel	5	8	6
Strobel et al. (2012)	BCP	1.6	2, 4, and 6	6
Kisiel et al. (2013)	Hyaluronan hydrogel/Fibronectin fragments	4	7	6
Ma et al. (2014)	BCP	20	8	6
Mumcuoglu et al. (2018)	Collagen-based microspheres/Alginate	0.3, 1, and 10	10	8
Lin et al. (2019)	Coralline HA	20	5	6
**Rat Calvarial Defect Model**				
**Author (Year)**	**Carrier**	**BMP Dose (** **µg)**	**Time** **(Weeks)**	**Sample Size (*n*)**
Jung et al. (2006)	TCP	2.5	2 and 8	20
Kim et al. (2011)	BCP	50 and 250	2 and 8	20
Park et al. (2011)	BCP	2.5	2 and 8	5–8
Notodihardjo et al. (2011)	HA	10	4	5
Jang et al. (2012)	BCP	2.5, 5, 10, and 20	2 and 8	8
Lee JH et al. (2013)	TCP, HA, BCP	5	4 and 8	13
Bae et al. (2017)	PCL/TCP	5	4	7
**Rat Femoral Defect Model**				
**Author (Year)**	**Carrier**	**BMP Dose (** **µg)**	**Time (Weeks)**	**Sample Size (*n*)**
Chu et al. (2006)	Poly(propylene fumarate)/TCP/DCP	10	6 and 15	4, 7
Johnson et al. (2011)	Collagen, Collagen/Heparin, Heparin	3	12	7, 9
Diab et al. (2011)	PCL/Silk fibroin hydrogel	5	12	10
Lee et al. (2012)	BCP	1000	4 and 8	6
Rodriguez-Evora et al. (2013)	Segmented polyurethane/PLGA/ TCP ceramics	1.6 and 6.5	12	9
Wai-Ching et al. (2014)	Bioactive glass/DCP	10	15	8, 9
Williams et al. (2015)	Collagen	25, 50, 75 and 100	8	8, 11
Krishnan et al. (2015)	Nanofiber mesh alginate	5 µg	12	14

**Table 3:** Rabbit segmental defect model.

Rabbit Segmental Defect Model					
Author (Year)	Model	Carrier	BMP Dose (µg)	Time (Weeks)	Sample Size (*n*)
Yoneda et al. (2004)	Femur (1.5 cm)	PLA-DX-PEG/TCP	50	24	5
Yamamoto et al. (2006)	Ulna (2 cm)	Gelatin hydrogel	17	6	3
Liu et al. (2009)	Radius (1.5 cm)	Gelatin/nanoHA/Fibrin	100	4, 8, and 12	5
Luca et al. (2010)	Radius (1.5 cm)	Chitosan hydrogel/TCP	150	8	1 (pilot)
Zhu et al. (2010)	Radius	nanoHA		4, 8, and 12	10
Bae et al. (2011)	Ulna (1.5 cm)	PCL/fibrin	75	8	5
Fujita et al. (2011)	Ulna (2 cm)	Gelatin/TCP	17	4 and 8	6, 10
Sun-Woong et al. (2012)	Ulna (2 cm)	PCL	15	12	6
Hou et al. (2012)	Radius (1.5 cm)	Collagen, Collagen/Chitosan	50	2, 4, 8, and 12	3, 5
Choi et al. (2014)	Radius (2 cm)	Collagen, Fibrin glue	50	6 and 12	4
Wu et al. (2014)	Radius (1.5 cm)	CaP cement, Hydroxypropylmethyl cellulose/CaP cement	50	2, 4, 8, and 12	5
Yamamoto et al. (2015)	Ulna (2 cm)	Gelatin/TCP	17	6	6
Peng et al. (2016)	Femur (1 cm)	PEG-PLGA hydrogel	5, 10, and 20	12	6
Pan et al. (2017)	Femur (2 cm)	Bioglass/TCP	20	4 and 8	5
Kuroiwa et al. (2018)	Femur (2 cm)	TCP	50	12 and 24	10
Grgurevic et al. (2019)	Ulna (1.5 cm)	Autologous blood coagulum	25, 50, and 100	23	5
Huang et al. (2021)	Ulna (2 cm)	TCP	20	8	5

**Table 4:** Rabbit PLF model.

Rabbit PLF Model				
Author (Year)	Carrier	BMP Dose (µg)	Time (Weeks)	Sample Size (*n*)
Boden et al. (1995)	DBM, Biocoral/ Collagen	100 and 300	5	14–16
Itoh et al. (1999)	Collagen	10, 50, and 200	24	6
Louis-Ugbo et al. (2001)	BCP, Collagen/BCP	3000/mL	5	18
Jenis et al. (2002)	Collagen	-	3 and 12	8
Konishi et al. (2002)	Autograft/HA	200	2, 4, and 6	2–7
Suh et al. (2002)	Collagen/BCP, BCP	860	5	14
Minamide et al. (2003)	TCP cement, True bone ceramics, Collagen	100	3 and 6	5–10
Namikawa et al. (2005)	TCP/PLA-DX-PEG	7.5, 15, and 30	6	5
Valdes et al. (2007)	-		6	18
Dohzono et al. (2009)	TCP	5, 15, 50, and 150	4 and 8	5–8
Lee JW et al. (2011)	Heparin—conjugated PLGA nanospheres, PLGA nanospheres	20	12	12
Lee JH et al. (2012)	HA	10, 50, 200, and 500	3 and 6	14
Vukicevic et al. (2019)	Autologous blood coagulum, Autologous blood coagulum/Allograft	125, 250, 500, and 1000	14	4

**Table 5:** Dog and sheep segmental defect model.

Dog Segmental Defect Model					
Author (Year)	Model	Carrier	BMP Dose (µg)	Time (Weeks)	Sample Size (*n*)
Itoh (1998)	Ulna (2 cm)	PLGA/Gelatin	40, 160, and 640	16	4
Tuominen (2000)	Ulna (2 cm)	Coral	-	16 and 36	3, 6
Hu (2003)	Radius (2 cm)	HA/Collagen/PLA	-	24	6
Jones (2008)	Ulna (2.5 cm)	Collagen/Allograft, Collagen/BCP ceramics	210, 430, and 650	12	6
Harada (2012)	Ulna (2.5 cm)	TCP	35, 140, 560, and 2240	12	3
Minier (2014)	Ulna (2 cm)	CaP/Hydrogel	330	20	5
**Sheep Segmental Defect Model**					
**Author (Year)**	**Model**	**Carrier**	**BMP Dose (** **µ** **g)**	**Time (Weeks)**	**Sample Size (*n*)**
Den Boer et al. (2003)	Tibia (3 cm)	HA	2500	12	8
Pluhar et al. (2006)	Tibia (5 cm)	Carboxymethylcellulose/Bovine collagen, Collagen	3500	16	10
Reichert et al. (2012)	Tibia (3 cm)	mPCL-TCP	3500	12 and 52	8
Cipitria et al. (2013)	Tibia (3 cm)	mPCL-TCP	1750 and 3500	12	8
Lammens et al. (2020)	Tibia (3 and 4.5 cm)	CaP ceramics	344, 1500, and 3800	16	4, 8, 13

**Table 6:** Sheep and non-humane primate PLF models.

Sheep PLF Model				
Author (Year)	Carrier	BMP Dose (µg)	Time (Weeks)	Sample Size (*n*)
Pelletier et al. (2014)	TCP	1050, 3500, and 10,500	12	12
Toth et al. (2016)	Collagen/BCP, Collagen-ceramic sponge	750 and 1500/cm^3^	24	12–24
Grgurevic et al. (2020)	Autologous blood coagulum, Autologous blood coagulum/Allograft	500 and 1500	27	6–10
**NHP PLF Model**				
**Author (Year)**	**Carrier**	**BMP Dose (** **µ** **g)**	**Time (Weeks)**	**Sample Size (*n*)**
Boden et al. (1999)	BCP	6000, 9000, and 12,000	24	4–12
Suh et al. (2002)	Ceramic/Collagen	9000	24	4
Akamaru et al. (2003)	Collagen/BCP Collagen/Allograft	3000	24	6
